# Health Behaviors and Cancer Diagnosis Among Individuals with Pathogenic Variants Associated with Hereditary Breast and Ovarian Cancer or Lynch Syndrome

**DOI:** 10.3390/jpm16010006

**Published:** 2025-12-26

**Authors:** Mahesh Sarki, Günther Fink, Souria Aissaoui, Fulvia Brugnoletti, Nicole Bürki, Rossella Graffeo, Christian Monnerat, Manuela Rabaglio, Ursina Zürrer-Härdi, Pierre O. Chappuis, Karl Heinimann, Maria C. Katapodi

**Affiliations:** 1 Department of Clinical Research, University of Basel, 4055 Basel, Switzerland; mahesh.sarki@unibas.ch; 2 Swiss Tropical and Public Health Institute, University of Basel, 4123 Allschwil, Switzerland; guenther.fink@swisstph.ch; 3 Fribourg Breast Centre, 1752 Fribourg, Switzerland; souria.aissaoui@genesupport.ch; 4 Genesupport, The Breast Centre, Hirslanden Clinique de Grangettes, 1224 Geneva, Switzerland; 5 Unit of Oncogenetics, Division of Precision Oncology, University Hospitals of Geneva, 1205 Geneva, Switzerland; fulvia.brugnoletti@hug.ch (F.B.);; 6 Women’s Clinic, University Hospital Basel, 4031 Basel, Switzerland; nicole.buerki@usb.ch; 7 Oncology Institute of Southern Switzerland, 6500 Bellinzona, Switzerland; rossella.graffeogalbiati@eoc.ch; 8 Department of Medical Oncology, Hospital of Jura, 2800 Delemont, Switzerland; christian.monnerat@h-ju.ch; 9 Department of Medical Oncology, Inselspital, Bern University Hospital, 3010 Bern, Switzerland; manuela.rabaglio@insel.ch; 10 Department of Medical Oncology, Cantonal Hospital Winterthur, 8400 Winterthur, Switzerland; ursina.zuerrer@ksw.ch; 11 Division of Genetic Medicine, University Hospitals of Geneva, 1205 Geneva, Switzerland; 12 Institute for Medical Genetics and Pathology, University Hospital Basel, 4031 Basel, Switzerland; karl.heinimann@usb.ch; 13 Research Group Human Genomics, Department of Biomedicine, University of Basel, 4031 Basel, Switzerland

**Keywords:** alcohol intake, BMI, cancer diagnosis, hereditary cancer syndromes, physical activity, smoking

## Abstract

**Background/Objectives:** Individuals carrying pathogenic/likely pathogenic (P/LP) variants associated with hereditary breast and ovarian cancer (HBOC) and Lynch Syndrome (LS)- have increased risk for various types of cancer. The study compared health behaviors, i.e., smoking, alcohol consumption, level of physical activity, and body mass index (BMI) among affected and unaffected (never diagnosed) individuals with P/LP variants associated with HBOC or LS. **Methods**: We used baseline and 18-month follow-up data from individuals with HBOC- or LS-associated P/LP variants from the Swiss CASCADE study, an open-ended, prospective, family-based cohort. Generalized linear models with random effects were applied. **Results:** A total of 856 records from 518 participants (HBOC: 410; LS: 108) were analyzed. More than half (58%) of participants had at least one cancer diagnosis. After controlling for potential confounders, the proportion of current smokers was not significantly different between the two groups (ß = 3.5, *p* = 0.24). Alcohol intake was not associated with cancer diagnosis (adjusted: ß = −0.2, *p* = 0.57), although it was positively associated with time since genetic testing (ß = 0.11, *p* < 0.01). Levels of physical activity were lower among affected individuals compared to unaffected (adjusted: ß = −0.5, *p* = 0.03). There was no difference in BMI between the two groups. **Conclusions:** No significant differences in health behaviors, i.e., smoking, alcohol consumption, or BMI, were detected in individuals with P/LP variants associated with HBOC or LS unaffected by cancer and those with cancer diagnosis. Lower levels of physical activity in those with a cancer diagnosis could potentially be attributed to cancer treatment. Future studies should examine whether adjustments in health behavior are associated with the genetic diagnosis.

## 1. Introduction

A cancer diagnosis can serve as a pivotal moment that can potentially trigger positive changes in health behaviors [1,2,3,4]. Studies show that many cancer survivors quit smoking [5,6], reduce alcohol consumption [6], increase their levels of physical activity [6], and maintain a body weight within normal range [5,7]. These changes in health behaviors are consistent with the 2012 American Cancer Society (ACS) Guidelines on Nutrition and Physical Activity for Cancer Survivors [8]. However, ACS guidelines do not specifically address the needs of individuals with a genetic predisposition to cancer, such as hereditary breast and ovarian cancer (HBOC) or Lynch syndrome (LS). HBOC is primarily associated with pathogenic/likely pathogenic (P/LP) variants in high penetrance genes, such as *BRCA1* and *BRCA2* (hereafter *BRCA*) [9]. LS is associated with P/LP variants in DNA mismatch repair genes (*MLH1, MSH2, MSH6*—hereafter MMR genes) and germline *EPCAM* deletions [10]. LS is associated with increased risk of colorectal cancer, and cancers of the endometrium, stomach, urinary tract, and skin [11]. HBOC and LS are monogenic disorders with autosomal dominant mode of inheritance, and they are actionable (e.g., early detection through imaging, risk-reducing surgeries, cascade testing for at-risk biological relatives) [12]. Both syndromes share some common characteristics, including the early age of onset and the potential for multiple cancer diagnoses. Individuals with germline pathogenic variants in *BRCA* or MMR genes have up to 20-fold higher risk for various types of cancer by the age of 70 years compared to the general population [13,14,15,16,17,18]. HBOC and LS individuals with a primary cancer diagnosis also have 2-fold higher risk of cancer recurrence [19,20].

A cancer diagnosis in individuals with HBOC or LS is primarily attributed to the germline predisposition. However, cancer risk is highly variable among these individuals, and little is known about why some develop cancer and others do not, and why some develop multiple cancers during their lifetime. Evidence indicates that germline P/LP variants may interact with somatic alterations to drive carcinogenesis, and this association has been reported for breast cancer [21,22,23,24]. This could suggest a possible link between health behaviors and cancer onset or a second cancer diagnosis. Cancer risk may increase due to behaviors that contribute to DNA damage, which in turn, are potentially associated with multiple cancer diagnosis [25,26,27]. The adoption of healthier behaviors may help maintain genome integrity, which helps sustain the function of the single copy of the wild-type allele. In turn, this could potentially prevent or delay cancer onset in individuals with germline P/LP variants associated with HBOC or LS [28]. Studies have shown that among carriers of P/LP variants in *BRCA* or MMR genes, those who smoke or have higher BMI have significantly higher risk for breast and colorectal cancer, respectively [27,29,30,31]. Conversely, cancer risk in these individuals is negatively associated with higher levels of physical activity [27,29,32,33,34,35]. Others reported that intake of more than 28 g of alcohol per day is associated with risk of colorectal cancer among individuals with LS [36,37]. However, no such association was found among individuals with P/LP variants in *BRCA* genes [38].

Little is known about changes in health behaviors among individuals with P/LP variants associated with HBOC or LS in relation to a cancer diagnosis. Inconsistency in previous reports could be due to study design, involving retrospective cohorts or case–control studies assessing health behaviors based on recall from the distant past [31,32,33], which is prone to recall bias [30,34,35,39]. The purpose of this study was to address this knowledge gap along with limitations related to retrospective study design. In individuals with HBOC or LS, cancer risk is mainly attributed to P/LP variants. However, modifiable risk factors may help preserve the function of the single copy wild-type allele, making a healthier lifestyle helpful in preserving genome integrity. Thus, it is essential to monitor modifiable risk factors related to health behaviors among individuals with P/LP variants in genes associated with cancer and contribute evidence to our knowledge base. We examined health behaviors in individuals with HBOC- or LS-associated variants, and compared smoking, alcohol consumption, level of physical activity, and BMI (as a proxy for dietary habits) between unaffected individuals (never diagnosed with cancer) and individuals with at least one cancer diagnosis associated with HBOC or LS. From a theoretical point of view, a cancer diagnosis may introduce a “teachable moment”, that is to create a unique window, where individuals are naturally more open to behavior change [40]. A cancer diagnosis in the context of genetic predisposition is a teachable moment because it increases emotional arousal, heightens risk awareness, and prompts reevaluation of priorities and habits [41]. It has been suggested that lifestyle interventions in HBOC and LS should be generally introduced after a cancer diagnosis, where motivation for behavioral changes is usually high [42,43].

## 2. Materials and Methods

This analysis is part of the Swiss CASCADE cohort (NCT03124212) [44], a family-based, multi-center, prospective study that targets adults (>18 years old) who live in Switzerland and have a confirmed diagnosis of carrying a P/LP variant associated with HBOC or LS through genetic testing. The cohort also recruits their biological relatives who may or may not carry the familial genetic predisposition, and they may or may not have a cancer diagnosis [44]. In order to be included in the cohort, participants must submit at least one questionnaire. The study has been approved by appropriate ethics committees (BASEC 2016-02052).

Recruitment of index cases (first member in the family identified with the P/LP variant) takes place in eight oncology/genetic testing centers in Switzerland. Index cases are asked to invite their biological relatives to the cohort. Participants complete a self-administered questionnaire at enrollment (baseline) available in local languages (German, French, Italian, or English). Follow-up questionnaires are administered approximately every 18–24 months. Two reminders, each after a 6-week interval, encourage participants to complete and return their questionnaire. Participants who do not return their questionnaire after the second reminder are considered lost to follow-up. No incentives are offered for participating in the cohort; however, one participant is randomly chosen annually to receive a gift card worth CHF 300 (approximately USD 325).

This analysis focuses on individuals with P/LP variants associated with HBOC or LS who have provided data one or two times between September 2017 and March 2025, depending on the time of enrollment in the cohort, irrespective of whether they were recruited as index cases or as relatives. Respondents were asked in each questionnaire if they ever had genetic testing and what was the test result. Relatives who had genetic testing but did not disclose their results were excluded from this analysis, if no medical record was available.

### 2.1. Primary Exposure Variables

Every questionnaire asked participants to indicate if they ever had a cancer diagnosis (“Yes”/“No”), and if “Yes”, the specific type of cancer diagnosis, and the age of diagnosis. Time since cancer diagnosis was calculated for each participant as a continuous variable, while the value was set to 0 for unaffected individuals.

### 2.2. Study Outcomes

Questionnaires asked participants to report their health behaviors during the past 4 weeks, in order to reduce recall bias and increase accuracy of reporting.

Smoking: Smoking status was coded as a categorical variable (“yes” coded as 1 and “no” coded as 0). Current smokers were asked to report the number of cigarettes smoked per day, which was multiplied by 7 to calculate number of cigarettes smoked per week, while never and non-current smokers were given a value of 0 for the average number of cigarettes smoked per week (continuous variable).

Alcohol: Alcohol consumption was calculated both as a categorical and as a continuous variable. Participants who reported never consuming alcohol were categorized as “never” drinkers (coded as 0); those consuming alcohol 1 to 2 days per week were categorized as “light” drinkers (coded as 1); those consuming alcohol 3 to 5 days per week were categorized as “moderate” drinkers (coded as 2); and those consuming alcohol 6 or 7 days per week were categorized as “heavy” drinkers (coded as 3) (categorical variable). An estimation of the average number of alcoholic beverages consumed per week was calculated by multiplying the group number (“never”−0, “light”−1, “moderate”−2, or “heavy”−3) with the average number of alcoholic beverages reported in each questionnaire (continuous variable).

Physical Activity: Participants who reported never engaging in physical activity were categorized as “no” exercisers (coded as 0); those engaging in physical activity once per week were categorized as “light” exercisers (coded as 1); those engaging in physical activity 2 to 3 days per week were categorized as “moderate” exercisers (coded as 2); and those engaging in physical activity 4 or more days per week were categorized as “heavy” exercisers (coded as 3) (categorical variable). An estimation of the total number of hours spent in physical activity per week was calculated by multiplying the group value (“never”−0, “light”−1, “moderate”−2, or “heavy”−3) with the average number of minutes of physical activity reported in each questionnaire (continuous variable).

BMI: Participants provided their body weight in kilograms and height in centimeters in each questionnaire. BMI was calculated as a continuous variable by dividing weight in kilograms by the square of the height in meters [45]. Participants were also categorized as “underweight” (<18.5 kgm^−2^) (coded as 0); “normal weight” (18.5−24.9 kgm^−2^) (coded as 1); “overweight” (25 to 29.9 kgm^−2^) (coded as 2); and “obese” (≥30 kgm^−2^) (coded as 3) (categorical variable).

In addition to age and sex, participants also reported their age at genetic testing and their age at first cancer diagnosis. Time since genetic testing and time since cancer diagnosis were computed by subtracting the date of genetic testing and the date of first cancer diagnosis from the date of questionnaire submission. Education level of participants was categorized as less than 12 years of education (coded as 0) vs. 12 or higher years of education (coded as 1). Participants were also asked whether they have ever received a diagnosis of depression from a healthcare provider (“yes” coded as 1 or “no” coded as 0).

### 2.3. Statistical Analyses

All analyses were conducted using the R statistical software package (R version 4.4.0). Descriptive statistics include calculation of means and standard deviations, medians and range, and frequencies and proportions. Although there was less than 5% missing data, analyses were carried out only in individuals with complete data. Two proportions z-tests were applied to compare frequencies and proportions. The non-parametric Wilcoxon rank sum test was used for comparing medians of variables that did not follow a normal distribution. Statistical significance was defined as *p*-values < 0.05.

For categorical outcomes (e.g., current smoking: yes or no), we applied a generalized linear mixed model, while for multi-group outcomes (e.g., alcohol: never, light, moderate and heavy), we applied cumulative link mixed models. Individual IDs were incorporated as random effects (to account for multiple observations recorded for some individuals) as observations were not independent. Also, a sensitivity analysis was conducted using clustered standard errors. Similarly, for each continuous variable (i.e., number of cigarettes per week; number of alcoholic drinks per week; hours spent in physical activity per week; and BMI), a linear mixed model was run with random effects of individual ID. In all random effect models, common independent variables included were age, sex, syndrome, cancer diagnosis (yes/no), and time since genetic testing. Model fit was assessed using Akaike Information Criterion (AIC) values for all models [46]. Finally, we examined the interaction effects of sex and time since cancer diagnosis, as well as sex and time since genetic testing on each outcome.

## 3. Results

**Figure 1:**
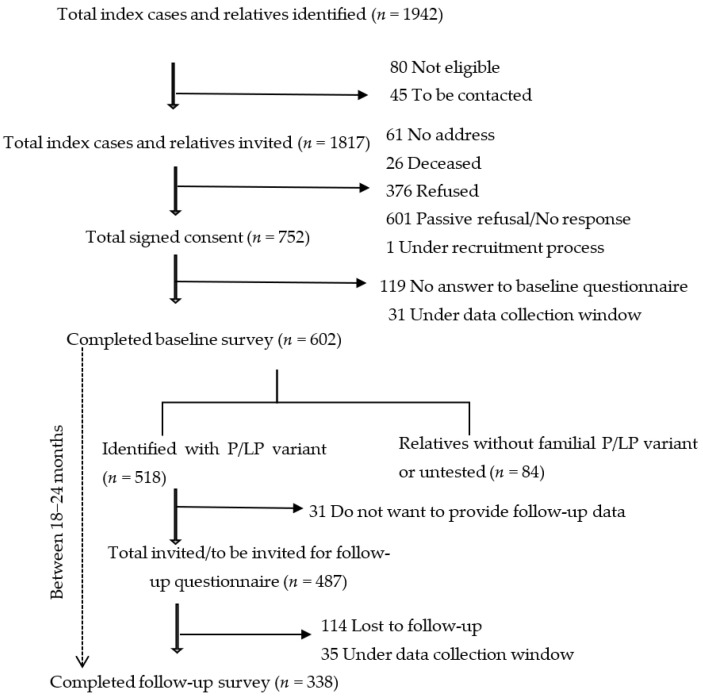
Study participants who completed baseline and/or follow-up questionnaires.

By March 2025, the study included 518 individuals with HBOC- or LS-associated P/LP variants who have provided baseline data. Among them 338 participants have also provided follow-up data, making for a total of 856 observations included in this analysis. Among the 518 individuals, 31 did not consent to providing follow-up data, 35 were in the window of providing follow-up data, while 114 were lost to follow-up (Figure 1). Common reasons for lost to follow-up were becoming critically ill, having an invalid address/moved abroad, and passive refusal. The 145 participants who did not provide follow-up data had similar rates of cancer diagnosis (63.4% vs. 57.3%, *p* = 0.21) and sex distribution (female: 81.7% vs. 81.7%, *p* = 0.26), but were significantly younger compared to those who did (48.72 ± 13.94 years vs. 51.32 ± 13.09 years, *p* < 0.01).

More than half of observations (58%) were from individuals with one or more previous cancer diagnosis, with a median of 6 years since the first diagnosis. Median time since genetic testing was 3.41 years. Most participants were female (81.7%), with the majority (79.4%) carrying HBOC-associated P/LP variants. A larger proportion of males were unaffected (never diagnosed) compared to females (57.3% vs. 38.2%, *p* < 0.01). Unaffected individuals were significantly younger compared to those with one or more cancer diagnosis (45.8 years old vs. 55.3 years old, *p* < 0.01). Between the baseline and the follow-up questionnaire 15 participants had a new cancer diagnosis; 5 of them had no cancer diagnosis at baseline, and 10 developed a second primary cancer. About 4% (*n* = 22) of individuals with cancer had genetic testing before cancer diagnosis.

The proportion of current smokers (13.7% vs. 11.2%, *p* = 0.07) and the average number of cigarettes smoked per week between the two groups was similar (Table 1). Univariate and multivariate analyses showed no significant association between smoking status or number of cigarettes smoked per week with cancer diagnosis, age, sex, syndrome, or time since genetic testing (Table 2 and Table 3).

**Figure 2:**
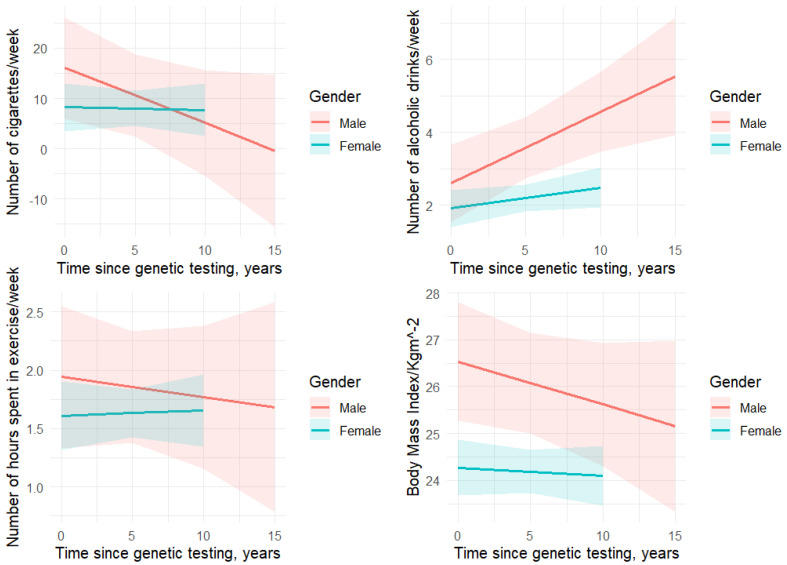
Interaction effect of gender and time since genetic testing on health behaviors.

There was a significantly higher proportion of “never” consume alcohol reporting among individuals with cancer compared to unaffected individuals (Table 1). In contrast, the proportion of “light” drinking (1–2 days/week) and “moderate” drinking observations was significantly higher among unaffected individuals compared to those with cancer (Table 1). No difference was observed in proportion of “heavy” drinking observations between the two groups. Cancer diagnosis was not associated with alcohol intake in both the univariate and adjusted models (univariate: ß = −0.2, *p* = 0.49; adjusted: ß = −0.2, *p* = 0.57) (Table 2 and Table 3). Females had lower alcohol intake than males (ß = −1.2, *p* < 0.01) (Table 3). There was a significant positive association between average number of alcoholic beverages consumed per week and time since genetic testing (ß = 0.11, *p* < 0.01) (Table 3). When we examined the interaction effect of sex with time since genetic testing on alcohol consumption, we found that with each time unit increase since genetic testing, males were 0.2 times more likely to report drinking alcohol (ß = 0.2, *p* = 0.01) (Figure 2).

The proportion of “moderate” exercise observations, i.e., engaging in physical activity 2–3 days per week, and the median number of hours engaging in physical activity per week were significantly higher among unaffected individuals compared to those with cancer (Table 1). The unadjusted and adjusted models showed that unaffected individuals were significantly more likely to engage in more hours of physical activity per week compared to those with cancer (unadjusted: ß = −0.37, *p* = 0.03; adjusted: ß = −0.5, *p* = 0.03) (Table 2 and Table 3).

Approximately 36% of the sample were overweight or obese. The proportion of “obese” observations was significantly lower among unaffected individuals compared to those with cancer (7.8% vs. 12.6%, *p* = 0.03), although there was not a significant difference in the median BMI between the two groups (Table 1). In both the unadjusted and adjusted models, cancer diagnosis was not significantly associated with BMI (Table 2 and Table 3). Age was positively correlated with BMI (ß = 0.04, *p* = 0.01) and females were more likely to have lower BMI compared to males (ß = −1.8, *p* < 0.01) (Table 3).

Diagnosis of depression showed no association with any health behavior in our sample, maybe because less than 10% of the sample indicated such a diagnosis in our sample (Table 3). We found no interactions between sex and time since cancer diagnosis for any of the outcome variables. The finding is presented in Supplementary Table S1. Sensitivity analyses showed that gender differences in alcohol intake and BMI appeared only in HBOC (Supplementary Table S2).

## 4. Discussion

We used longitudinal data from a prospective cohort to compare health behaviors in a sample of individuals with and without a cancer diagnosis carrying P/LP variants associate with HBOC or LS. One or more cancer diagnoses is associated only with changes in physical activity in our sample, which is inconsistent with a cohort study with LS-carriers from Finland, which reported positive associations between cancer diagnosis and health behaviors [35]. The inconsistent findings may be explained either by the retrospective design in the Finnish study and reporting of health behaviors from the distant past, or to the more diverse sample of our study that also included individuals with P/LP variants associated with HBOC, with an overrepresentation of female carriers.

We found that individuals with P/LP variants associated with HBOC or LS with one or more cancer diagnosis exercise fewer hours per week compared to their unaffected counterparts. Decreased levels of physical activity in cancer survivors could be attributed to decreased functional status and/or increased levels of fatigue or other persistent symptoms, attributed to cancer treatment and its side effects [47,48]. A recent meta-analysis reported that up to 42% of cancer survivors experience significantly higher levels of fatigue compared to healthy controls [49]. In our sample, the median time since first cancer diagnosis was 6 years, which may explain the lower level of engagement in physical activity among individuals with one or more cancer diagnosis. However, it is also important to note that in our sample the median time engaging in physical activity among individuals with cancer was 1 h (60 min) per week, which is significantly less compared to 150 min per week, as recommended by the international guidelines such as ACS [8]. This is an important finding, given that physical activity may delay the onset of cancer recurrence in individuals with HBOC or LS [34,50], by improving mobilization and activation of mucosal immune cells [51,52], improving metabolic signaling and reducing systemic low-grade inflammation that creates a permissive micro-environment for tumor growth [52] and reducing adipose-driven estrogen production that may promote hormone-sensitive tumor growth [53]. Given the documented benefits of physical activity for cancer survivors [54,55,56], efforts should focus on establishing a systematic integration of physical activity into cancer survivorship programs, especially for individuals with HBOC or LS. Research should also focus on uncovering the effects of physical activity on mechanisms of carcinogenesis among individuals with HBOC or LS, which may be different from those in sporadic cancer.

There was no statistically significant difference in smoking, alcohol intake, and BMI between individuals with HBOC or LS with and without a cancer diagnosis. However, our sample included only 12% of current smokers, and there was not a significant difference in proportion of smoking observations between the two groups, a finding consistent with a previous study [38]. However, it is also important to note that current smoking among unaffected individuals in our sample was lower compared to the general Swiss adult population (14% vs. 25%) [57,58,59], suggesting that behavioral adjustments may be triggered by genetic testing and the detection of the P/LP variant. Studies report conflicting findings regarding the association between smoking and carcinogenesis in individuals with HBOC or LS. Observational studies reported no association between smoking and development of ovarian or colorectal cancer in HBOC or LS, respectively [60,61], while a machine learning modeling study reported a positive association between long-term smoking and breast carcinogenesis in carriers of P/LP variants in *BRCA* genes [62]. Given the known carcinogenic effects of smoking in the general population, and its effects on cancer recurrence [63], offering smoking cessation support to individuals with a genetic predisposition to cancer could have a significant public health benefit.

Female participants reported lower levels of alcohol intake compared to males, a finding that has been reported for individuals with sporadic forms of cancer [6], but has not been confirmed for individuals with P/LP variants associated with HBOC or LS [37,38]. In our study, the proportion of participants reporting having at least one alcoholic drink per week and the average number of alcoholic drinks per week were lower compared to the general Swiss adult population (71% vs. 80% and 2.31 vs. 2.90 drinks, respectively) [58,64]. The positive association between alcohol intake with time since genetic testing may also indicate that changes in health behaviors can be triggered by genetic testing results rather than a cancer diagnosis. Importantly, we found a significant positive correlation between male and time since genetic testing, i.e., with each increase in time unit after genetic testing, male participants were 0.2 times more likely to report drinking alcohol. However, this gender difference is limited to HBOC participants only, where males constitute only a small portion of the HBOC group.

Female participants had a lower BMI compared to males, a finding that has also been reported for sporadic forms of cancer [65]. The proportion of obese observations in our sample was significantly higher among individuals with cancer, who were on average 10 years older compared to their unaffected counterparts, which is an expected finding given that BMI typically increases over the lifetime. Importantly, our study included a lower proportion of overall overweight or obese individuals compared to the general Swiss population of similar age range (35% vs. 42%) [58,66]. Gender differences in BMI should be interpretated with caution as this difference was observed only in HBOC and nearly 87% of the HBOC participants were females.

Compared to the general Swiss population, our study participants appeared to show healthier behaviors indicating that these may have changed upon receiving genetic testing results. However, since only 22 participants had genetic testing prior to their cancer diagnosis, it is not feasible to produce a reliable model to assess the temporal relationship between genetic testing, cancer diagnosis, and behavioral changes. It is also possible that behavioral changes motivated by a cancer diagnosis are not sustained due to potentially lack of structured, long-term clinical support for individuals with P/LP variants. Integrating health behavior support during follow-up visits with general practitioners could be key in promoting health behaviors and sustaining them for these individuals.

The main strength of the study includes the longitudinal design, where health behaviors and new cancer diagnoses were collected prospectively. Questionnaires asked participants to report their health behaviors during the past 4 weeks in order to reduce recall bias and increase accuracy. Data were handled using a longitudinal modeling approach. The CASCADE study is the largest cohort of individuals with hereditary cancer predisposition in Switzerland, with a representative sample. The combined analysis of HBOC and LS can also enhance the generalizability of findings for actionable genetic conditions. Finally, the study provides a comprehensive assessment of four common health behaviors and a relatively large number of observations. However, we cannot exclude the possibility of social desirability and self-selection biases associated with self-reported health assessments. Moreover, the 18−24-month follow-up period might be too short a time to capture behavior changes. Our sample was on average 3.5 years post genetic testing, and data to assess behavioral changes that occurred immediately after or close to the genetic diagnosis were not available. Time since genetic testing was included as a covariate, but addressing the effects of receiving a diagnosis of a genetic predisposition to cancer requires a separate analysis. Gender differences should be interpreted with caution, as these differences were observed only in HBOC, and males represented only about 13% of the HBOC participants. Participants who were lost to follow-up (22%) were younger, which may be an alternative explanation of the lower rates of smoking and alcohol consumption in the analyzed cohort than in the general Swiss population.

## 5. Conclusions

Studying health behaviors and risk for various types of cancer is essential. These associations have been reported for the general population based on large prospective longitudinal population-based cohorts. This type of large data is currently not available for HBOC or LS populations due to the lower prevalence of these syndromes, lack of standardized assessments for health behaviors, and lack of infrastructure for storage and sharing of routinely collected data. Although the probability of developing cancer among HBOC or LS populations is primarily driven by the pathogenic variant [17,67,68], it is possible that the beneficial or harmful effects of modifiable risk factors are exacerbated among these individuals. A healthier lifestyle could potentially help maintain genome integrity, which can prevent the occurrence of cancer [28]. Therefore, it is important to monitor these modifiable risk factors among individuals with P/LP variants, especially in high penetrance genes associated with HBOC or LS, while providing support when necessary for behavioral changes. The results of our study could be used to inform behavioral interventions for modifiable risk factors. Follow-up visits for cancer surveillance could include assessment of smoking, alcohol intake, physical activity, and BMI and promote tailored interventions for behavioral change among individuals with P/LP variants associated with cancer predisposition. Finally, there is a need for guidelines dedicated to these high-risk populations.

## Figures and Tables

**Table 1:** Observations from carriers of germline HBOC- and LS-associated P/LP variants with and without a cancer diagnosis.

	Overall Number of Observations *n* = 856 (%)	Observations from Individuals Never Diagnosed with Cancer *n* = 357 (%)	Observations from Individuals with at Least One Cancer Diagnosis ^a^ *n* = 499 (%)	*p*-Value
Demographic and clinical characteristics				
Sex				
Male	157 (18.3)	90 (25.2%)	67 (13.4%)	<0.01 ^b^
Female	699 (81.7)	267 (74.8%)	432 (86.6%)	<0.01 ^b^
Age [mean, (SD)], years	51.4 (13.1)	45.8 (13.1)	55.3 (11.4)	<0.01 ^c^
Education level [missing = 16]				
Twelve or higher years of education	733 (87.3)	330 (93.5)	403 (82.8)	<0.01
Syndrome				
HBOC	680 (79.4)	305 (85.4%)	375 (75.2%)	<0.01 ^b^
LS	176 (20.6)	52 (14.6%)	124 (24.8%)	<0.01 ^b^
Depression [missing = 65]				
Diagnosis of depression (yes)	71 (9.2)	25 (7.5)	48 (10.5)	0.19
Median time since genetic testing (Q1–Q3), years	3.41 (2.00–6.30)	3.00 (1.93–5.65)	3.73 (2.01–6.71)	<0.01 ^c^
Median number of cancer diagnosis (Q1–Q3), (range)	-	-	1 (1–1)	
Median time since first cancer diagnosis (Q1–Q3), years	-	-	6.00 (3.0–12.28)	
Health Behaviors				
Current smoking (missing = 3)				
Yes	105 (12.3)	49 (13.7%)	56 (11.2%)	0.07 ^b^
No	748 (87.4)	308 (86.3%)	440 (88.2%)	0.47 ^b^
Average number of cigarettes [mean, (SD)] smoked per week	72.24 (64.17)	51.90 (40.45)	90.7 (75.59)	0.31 ^d^
Alcohol (missing = 12)				
Never (0)	258 (30.1)	81 (22.7%)	177 (35.5%)	<0.01 ^b^
Light (1–2/week)	379 (44.3)	173 (48.5%)	206 (41.3%)	0.04 ^b^
Moderate (3–5/week)	130 (15.2)	65 (18.2%)	65 (13.0%)	0.05 ^b^
Heavy (≥6/week)	77 (9.0)	28 (7.8%)	49 (9.8%)	0.38 ^b^
Average number of alcoholic beverages [mean, (SD)] per week (missing = 12)	2.31 (3.77)	2.42 (3.59)	2.23 (3.89)	0.47 ^d^
Physical activity (missing = 12)				
No exercise (0)	97 (11.3)	31 (8.7%)	66 (13.2%)	0.05 ^b^
Light exercise (1/week)	240 (28.0)	97 (27.2%)	143 (28.7%)	0.69 ^b^
Moderate exercise (2–3/week)	358 (41.8)	171 (47.9%)	187 (37.5%)	<0.01 ^b^
Heavy exercise (≥4/week)	149 (17.4)	56 (15.7%)	93 (18.6%)	0.30 ^b^
Median hours of physical activity (IQR ^e^) per week (missing = 55)	1.00 (0.3–2.0)	1.0 (0.5–2.0)	1.0 (0.25–2.0)	0.05 ^c^
Body Mass Index (BMI)				
Underweight (<18.5 kgm^−2^),	30 (3.5)	10 (2.8%)	20 (4.0%)	0.44 ^b^
Normal weight (18.5–24.9 kgm^−2^)	498 (58.2)	211 (59.1%)	287 (57.5%)	0.69 ^b^
Overweight (25.0–29.9 kgm^−2^)	215 (25.1)	97 (27.2%)	118 (23.6%)	0.27 ^b^
Obese (≥30 kgm−^2^)	91 (10.6)	28 (7.8%)	63 (12.6%)	0.03 ^b^
Median BMI (Q1-Q3) (missing = 22), kgm^−2^	23.6 (21.3–27.1)	23.4 (21.3–26.5)	23.9 (21.2–27.4)	0.43 ^c^

^a^: any cancer diagnosis (brain, lung, liver, small intestine, colon, rectal, kidney, urinary tract, prostate, breast invasive, ductal carcinoma in situ, ovarian, uterus, cervix, fallopian tubes, thyroid, non-Hodgkin lymphoma, and multiple myeloma); ^b^: two proportion Z-test; ^c^: Wilcoxon rank sum test; ^d^: *t*-test; ^e^ IQR = Interquartile Range (Q1–Q3).

**Table 2:** Univariate models with random effects of ID variable.

	Smoking					Alcohol					Physical Activity					Body Mass Index (BMI)				
	Current Smoking Status (Yes or No) ^a^		Average Number Cigarettes Smoked per Week ^c^			Alcohol Intake Group (Never, Light, Moderate, Heavy) ^b^		Average Number Alcoholic Beverages per Week ^c^			Physical Activity Group (No Exercise, Light, Moderate, Heavy) ^b^		Average Hours Physical Activity per Week ^c^			BMI (Underweight, Normal Weight, Overweight, Obese) ^b^		BMI ^c^		
	OR	95% CI	Est.	Std.er.	*p*	OR	95% CI	Est.	Std.er.	*p*	OR	95% CI	Est.	Std.er.	*p*	OR	95% CI	Est.	Std.er.	*p*
Cancer diagnosis	0.8	0.10–6.0	2.4	2.8	0.38	0.5	0.2–1.3	−0.2	0.3	0.49	0.81	0.6–1.1	−0.37	0.17	0.03	0.8	0.2–3.3	0.4	0.3	0.3

^a^: Generalized mixed model; ^b^: Cumulative link mixed model, ^c^: Linear mixed model.

**Table 3:** Multivariate models with random effects of ID variable.

	Smoking					Alcohol					Physical Activity					Body Mass Index (BMI)				
	Current Smoking Status (Yes or No) ^a,d^		Average Number Cigarettes Smoked per Week ^c,e^			Alcohol Intake Group (Never, Light, Moderate, Heavy) ^b,f^		Average Number Alcoholic Beverages per Week ^c,g^			Physical Activity Group (No Exercise, Light, Moderate, Heavy) ^b,h^		Average Hours Physical Activity per Week ^c,i^			BMI Group (Underweight, Normal Weight, Overweight, Obese) ^b,j^		BMI ^c,k^		
	OR	95% CI	Est.	Std.er.	*p*	OR	95% CI	Est.	Std.er.	*p*	OR	95% CI	Est.	Std.er.	*p*	OR	95% CI	Est.	Std.er.	*p*
Cancer diagnosis	0.3	0.02–4.2	3.5	3.0	0.24	0.41	0.12–1.42	−0.2	0.3	0.57	0.7	0.5–1.1	−0.5	0.2	0.03	0.83	0.32–2.13	0.2	0.4	0.64
Age in years	1.02	0.9–1.1	−0.1	0.11	0.35	1.03	0.98–1.08	0.03	0.01	0.02	1.02	1.0–1.03	0.01	0.01	0.11	0.93	0.92–0.93	0.04	0.02	0.01
Female (ref: male)	0.5	0.03–6.7	−4.5	3.8	0.24	0.51	0.11–2.35	−1.2	0.4	<0.01	1.1	0.6–1.8	−0.01	0.3	0.97	-	-	−1.8	0.6	<0.01
LS (ref: HBOC)	2.9	0.2–43.8	3.4	3.5	0.33	0.65	0.14–3.05	−0.6	0.4	0.14	0.8	0.5–1.3	0.3	0.2	0.97	-	-	−0.4	0.5	0.40
Education (≥12 years education)	0.5	0.01–16.6	−7.8	4.2	0.06	0.74	0.13–4.29	−0.2	0.5	0.63	1.3	0.7–2.5	0.3	0.3	0.28			−1.1	0.6	0.09
Diagnosis of depression	0.2	0.02–33.8	−0.7	3.2	0.82	0.40	0.01–1.90	0.2	0.4	0.67	1.0	0.5–1.9	−0.2	0.3	0.53			0.02	0.3	0.96
Time since genetic testing	1.00	0.8–1.2	−0.1	0.3	0.80	1.12	0.97–1.29	0.11	0.03	<0.01	1.0	0.95–1.04	−0.01	0.02	0.73	-	-	−0.01	0.04	0.93

^a^: Generalized mixed model; ^b^: Cumulative link mixed model, ^c^: Linear mixed model. AIC values: ^d:^ 314.8; ^e^: 6651.6; ^f^: 1493.4; ^g^: 3644.7; ^h^: 1784.4; ^i:^ 3110.1; ^j^: 1041.8; ^k^: 3522.6.

## Data Availability

The CASCADE Consortium is open to collaboration with national and international researchers. Interested parties can contact the PI to discuss project ideas and access to data. Decisions are made in collaboration with the Scientific Board. Templates for data requests are available at https://swisscascade.ch/en/research-project-data-request/ (accessed on 15 November 2025).

## Supplementary Materials

The following supporting information can be downloaded at: https://www.mdpi.com/article/10.3390/jpm16010006/s1, Table S1: Multivariate linear mixed models of the interaction effect of sex with time since cancer diagnosis. Table S2: The association between cancer diagnosis and health behaviors, segregated by syndrome.
