# Supplementary material for: Health Behaviors and Cancer Diagnosis Among Individuals with Pathogenic Variants Associated with Hereditary Breast and Ovarian Cancer or Lynch Syndrome
Source: J Pers Med. 2025 Dec 26;16(1):6. doi: 10.3390/jpm16010006 (PMC12843232; doi:10.3390/jpm16010006)
Supplement: Supplementary file 1 [file jpm-16-00006-s001.zip › jpm-3918252-supplementary.pdf]

Supplementary Table S1: Multivariate [a] linear mixed models of the interaction effect of sex with time since cancer diagnosis.

	**Average number of cigarettes smoked per week**			**Average number of alcoholic beverages per week**			**Average hours of physical activity per week**			**Body Mass Index**		
	Est.	Std.er.	*p*	Est.	Std.er.	*p*	Est.	Std.er.	*p*	Est.	Std.er.	*p*
Female (ref: male)	-4.5	4.8	0.35	-1.6	0.5	0.01	-0.4	0.3	0.12	-2.0	0.6	<0.01
Time since cancer diagnosis	-0.28	0.3	0.38	-0.04	0.05	0.37	-0.006	0.03	0.11	-0.03	0.06	0.65
Female * time since cancer diagnosis	0.47	0.5	0.39	-0.01	0.06	0.26	0.05	0.03	0.09	0.01	0.07	0.83

[a]: Model adjusted for age, syndrome and time since genetic testing

Supplementary Table S2: The association between cancer diagnosis and health behaviors, segregated by syndrome

	**Average number of cigarettes smoked per week**						**Average number of alcoholic beverages per week**						**Average hours of physical activity per week**						**Body Mass Index (BMI)**					
	HBOC			LS			HBOC			LS			HBOC			LS			HBOC			LS		
	Est	Std Er.	*p*	Est	Std Er.	*p*	Est	Std Er.	*p*	Est	Std Er.	*p*	Est	Std Er.	*p*	Est	Std Er.	*p*	Est	Std Er.	*p*	Est	Std Er.	*p*
Cancer diagnosis	1.6	2.9	0.59	12.6	9.4	0.18	-0.3	0.35	0.45	0.31	0.88	0.73	-0.34	0.22	0.11	-0.93	0.65	0.15	-0.03	0.45	0.94	2.02	1.76	0.09
Age in years	-0.02	0.12	0.84	-0.44	0.30	0.15	0.04	0.01	<0.01	-0.01	0.03	0.77	0.01	0.01	012	0.01	0.02	0.72	0.05	0.02	0.01	-0.004	0.04	0.91
Female (ref: male)	-5.49	4.4	0.21	-3.88	7.95	0.63	-1.45	0.51	<0.01	-0.82	0.75	0.28	-0.27	0.31	0.39	0.34	0.57	0.55	-1.88	0.71	0.01	-1.57	0.99	0.12
Education level (12 years of education or higher)	-5.2	4.6	0.27	-12.5	9.85	0.21	-0.38	0.54	0.48	0.24	0.91	0.79	0.24	0.34	0.48	0.42	0.71	0.55	-1.58	0.75	0.04	0.62	1.23	0.62
Depression (ref: no)	-2.5	3.00	0.41	3.19	9.89	0.75	-0.15	0.48	0.76	1.61	0.98	0.11	-0.26	0.32	0.42	0.22	0.93	0.82	-0.03	0.32	0.93	0.09	0.58	0.88
Time since genetic testing	0.26	0.32	0.42	-0.42	0.60	0.48	0.14	0.04	<0.01	0.07	0.06	0.21	0.02	0.02	0.49	-0.04	0.04	0.38	-0.04	0.05	0.38	0.07	0.06	0.29
